# Histological and molecular features of the subacromial bursa of rotator cuff tears compared to non-tendon defects: a pilot study

**DOI:** 10.1186/s12891-021-04752-1

**Published:** 2021-10-14

**Authors:** Susann Minkwitz, Kathi Thiele, Aysha Schmock, Nicole Bormann, Thanh Huyen Nguyen, Philipp Moroder, Markus Scheibel, Britt Wildemann, Fabian Plachel, Franka Klatte-Schulz

**Affiliations:** 1 Julius Wolff Institute, Berlin Institute of Health at Charité – Universitätsmedizin Berlin, 13353 Berlin, Germany; 2 BIH-Center for Regenerative Therapies, Berlin Institute of Health at Charité – Universitätsmedizin Berlin, 13353 Berlin, Germany; 3 Center for Musculoskeletal Surgery, Charité-Universitaetsmedizin Berlin, 13353 Berlin, Germany; 4 Department Shoulder and Elbow Surgery, Schulthess Klinik, 8008 Zürich, Switzerland; 5 Experimental Trauma Surgery, Department of Trauma-, Hand- and Reconstructive Surgery, Jena University Hospital, Friedrich Schiller University Jena, 07747 Jena, Germany

**Keywords:** Subacromial bursa, Shoulder pathology, Tendon tear, Healing, Histology, Gene expression

## Abstract

**Background:**

The role of the subacromial bursa in the development or healing of shoulder pathologies is unclear. Due to this limited knowledge, we aimed to understand specific reactions of the subacromial bursa according to rotator cuff (RC) pathologies compared to non-tendon defects of the shoulder. We hypothesized that the tissue composition and inflammatory status of the bursa are likely to vary between shoulder pathologies depending on the presence and the extent of RC lesion.

**Method:**

Bursa samples from patients with either 1) shoulder instability with intact RC (healthy bursa, control), 2) osteochondral pathology with intact RC, 3) partial supraspinatus (SSP) tendon tear, or 4) full-thickness SSP tear were investigated histologically and on gene expression level.

**Result:**

Bursae from SSP tears differed from non-tendon pathologies by exhibiting increased chondral metaplasia and TGFβ1 expression. MMP1 was not expressed in healthy bursa controls, but strongly increased with full-thickness SSP tears. Additionally, the expression of the inflammatory mediators IL1β, IL6, and COX2 increased with the extent of SSP tear as shown by correlation analysis. In contrast, increased angiogenesis and nerve fibers as well as significantly upregulated IL6 and COX2 expression were features of bursae from patients with osteochondral pathology. Using immunohistochemistry, CD45+ leukocytes were observed in all examined groups, which were identified in particular as CD68+ monocytes/macrophages.

**Conclusion:**

In summary, besides the strong increase in MMP1 expression with SSP tear, molecular changes were minor between the investigated groups. However, expression of pro-inflammatory cytokines correlated with the severity of the SSP tear. Most pronounced tissue alterations occurred for the osteochondral pathology and full-thickness SSP tear group, which demonstrates that the bursal reaction is not exclusively dependent on the occurrence of an SSP tear rather than longstanding degenerative changes. The present bursa characterization contributes to the understanding of specific tissue alterations related to RC tears or non-tendon shoulder pathologies. This pilot study provides the basis for future studies elucidating the role of the subacromial bursa in the development or healing of shoulder pathologies.

## Background

The bursa is a fluid filled sac with a surrounding synovial membrane that functions in joints to reduce friction [1]. The subacromial bursa, the largest bursa in the shoulder, is located between the acromion and the supraspinatus tendon (SSP) of the rotator cuff (RC), allowing for frictionless shoulder movement. A narrowing of the subacromial space leads to subacromial pain and restricted movement. The subacromial bursa is often linked to shoulder pain, which is one of the most common complaints amongst musculoskeletal consultations in athletes as well as in the general population [2, 3]. Subacromial decompression, the removal of bone spurs and soft tissue including the bursa, is one of the most commonly performed shoulder surgeries. A recent study has claimed that this surgical procedure is ineffective and without benefit for the patients regarding pain and regain of joint function [4]. However, other findings support the value of arthroscopic subacromial decompression [5, 6]. It was hypothesized that a bursectomy might be beneficial due to the elimination of inflammatory cytokines and metalloproteases within the inflamed bursa [7, 8]. On the other hand, the benefits of retaining the bursa were linked to its importance as a source of cells and vasculature as well as immunomodulatory and growth factors for tendon healing [9–13]. Due to the lack of consensus in the field, many different clinical methods are used ranging from no or partial resection of bursal tissue to complete resection. Clarification of the role of the bursa in pathology and healing is of utmost clinical importance for the effective treatment of shoulder pathologies. Due to the potentially beneficial cellular composition as well as growth factor content, we speculate that the bursa may serve as a supportive structure to augment tendon healing after RC repair. An easy and reliable arthroscopic surgical technique has already been described in which the RC tendons are covered with the subacromial bursa, using the highly vascularized bursa to augment RC repair [14, 15]. Additionally, a recent technical note has reported on a method for re-implanting autologous minced bursa tissue obtained from bursectomy to the RC surface with the aim of supporting RC repair [16].

To date, bursae have received limited attention with only a few studies outlining a possible correlation between the histopathological findings observed in the bursa and the clinical symptoms of patients. Bursa tissue changes are caused by repetitive mechanical irritation of the bursa. The resulting inflammatory reaction to mechanical irritation can cause increased proliferation of fibroblasts, angiogenesis, and formation of type III collagen [1, 17]. The analysis of cytokines and matrix metalloproteinases (MMPs) in bursitis tissue from patients with RC tear or frozen shoulders showed an increased inflammatory reaction, whereas none, or only mild inflammatory evidence was present in healthy bursa tissue from patients with shoulder instability [7, 18] . Further studies demonstrated innervation in bursa tissue and its correlation with pain [9, 19]. From a cellular perspective, isolated bursa fibroblasts show clear stem cell potential with a stem cell phenotype [20] and a multipotent differentiation potential, which was comparable to bone marrow stem cells [21] or even better [10].

The aforementioned studies concentrated on the comparison of RC tears with intact controls, but knowledge regarding the bursal reaction according to osteochondral pathologies or differentiation between partial and full-thickness SSP tears or tear severity is missing. This comparison will help to understand specific alterations of the bursa tissue according to SSP tears and bordering it from reactions triggered by non-tendon shoulder pathologies. With this, we aim to contribute to an improved understanding of bursa tissue and to resolve some of the controversy in the field.

## Material and methods

### Tissue sampling

Samples of the subacromial bursa were harvested from the lateral subacromial site during shoulder surgery and subsequently characterized. Samples were grouped according to the RC lesion: Group 1: Healthy bursae from patients with macroscopically intact RC (shoulder or acromioclavicular (AC) joint instability). These patients had no history of dysfunction and shoulder pain, and no previous surgery on the affected shoulder. Group 2: Patients with a degenerative osteochondral pathology and macroscopically intact RC. Samples were taken during open surgery from patients who underwent surgical treatment for either humeral head necrosis or omarthrosis receiving arthroplasty. Group 3: Patients with a degenerative partial tear of the SSP; and Group 4: Patients with a degenerative full-thickness tear of the SSP. Samples from group 1, 3 and 4 were taken via the anterolateral portal in the subacromial space in the case of arthroscopic intervention. A standardized biopsy forceps was used to obtain the samples. The preferred site for specimen collection was far lateral, as this tissue can also be used for subsequent RC reconstruction and augmentation. Tear morphology was evaluated using the Patte classification for the extent of tendon retraction [22] and intraoperatively the tear size was classified according to Bayne and Bateman [23]. In case of open surgery in group 2, the arthrotomy was performed via a deltopectoral approach. By keeping the deltoid muscle away, direct access to the subacromial bursa was possible as an existing sliding layer between the acromion and the RC underneath. Demographic parameters of the groups are listed in Table 1. The samples were separated into two parts for histology and RNA isolation. Due to the limited size of the samples obtained, not all bursa samples underwent all histological and gene expression analyses. The study was approved by the local IRB (EA1/267/15).

**Table 1:** Demographic parameters of groups

	Group			
	1	2	3	4
**Pathology**	Shoulder / AC joint instability (healthy group)	Osteochondral pathology (Necrosis^a^/Arthrosis^b^)	Partial SSP tear	Full-thickness SSP tear
**RC lesion**	Macroscopically intact RC	Macroscopically intact RC	Partial SSP tear	Full-thickness SSP tear
**n**	5	7	6	16
**Age**	28.8 ± 7.6	64.7 ± 15.5	55.0 ± 9.4	59.0 ± 9.0
**BMI**	23.7 ± 4.4	26.9 ± 6.6	27.8 ± 6.8	26.0 ± 5.2
**Sex (f/m)**	1/4	4/3	1/5	5/11
**Patte score**	–	–	0: 6x	1: 9x; 2: 5x; 3: 2x
**Bateman score**	–	–	–	2: 11x; 3: 3x; 4: 2x
**Snyder classification**	–	–	A3: 1x; B1: 2x; B2: 1x; B3: 1x, intratend.: 1x	–

*AC* acromioclavicular

^a^one avascular, and one posttraumatic humeral head necrosis

^b^one grade 1 omarthrosis with humeral head fracture and four grade 3 omarthrosis

### Histology

Bursa samples were fixed in 4% PFA for 24 h and embedded in paraffin. Slices 4 μm thick were stained with Hematoxylin and Eosin (H&E, Chroma-Waldeck), Alcian Blue (Sigma Aldrich) for chondral metaplasia, the antibody against α-smooth muscle actin (αSMA) for blood vessels, the surface marker CD45 and CD68 for immune cells as well as S100 for nerve fibers. CD45 and CD68 are markers for relevant immune cell populations in bursa tissue and present investigations should determine possible differences between SSP tears and non-tendon pathologies. For immunohistochemistry the tissue was incubated with the primary antibodies against αSMA (monoclonal mouse anti-αSMA, 1:200, Dako, M0851), CD45 (monoclonal mouse anti-CD45, 1:100, Dako, M0701), CD68 (monoclonal mouse anti-human CD68, 1:40, Acris Antibodies GmbH), and S100 (1:400, mouse anti-S100, Zytomed, MSK050) for 1 h at room temperature. The staining was performed with the ZytoChem-Plus AP Kit (Broad Spectrum, AP060, Zytomed) and the Alkaline Phosphatase Substrate Kit I (Sk-5100, Vector) was used as the detection system. Slices were counterstained with Mayer’s Hematoxylin (Merck KGaA). Negative controls were stained using the secondary antibody only, as well as an isotope control staining. A histological 3-point evaluation system was established, adapting the histological scores by Chillemi et al. [24] and Krenn et al. [25] to examine the quality of the bursa tissue as shown in Table 2. Samples could be scored with a minimum of 9 to a maximum of 27 points. A high histological score defines a strong bursal reaction due to degenerative changes, whereas a low score indicates an unaffected healthy tissue. Three blinded independent observers performed the evaluation. The interobserver variability was evaluated by the interclass correlation coefficient (ICC) for each score parameter and was considered as high with a mean of 0.866 (0.803–0.918). The intraobserver variability showed a mean ICC of 0.939 (0.808–0.990).

**Table 2:** Classification of histological score

Characteristics	Staining	Classification		
		1	2	3
Disarray	H&E	Highly organized	Partially organized	Highly unorganized
Angiogenesis	α-SMA	Sporadic blood vessels	Several blood vessels	Numerous blood vessels in whole tissue
Fatty metaplasia	H&E	Absent	Focal	Extended
Hypertrophy of synovium	H&E	1 layer	2–3 layers	4–5 layers
Immune cells	CD45	Absent	Few immune cells mainly perivascular	Numerous immune cells inside the tissue
Accumulation of fibroblasts	H&E	Only fibrocytes (small, elongated nucleus)	Slightly increased number of fibroblasts	Greatly increased number of fibroblasts
Occurrence of villi	H&E	Absent	Focal	Extended
Chondral metaplasia	Alcian Blue	Absent	Focal	Extended
Nerve fibers	S100	Absent	Few nerve fibers	Several nerve fibers

### qRT-PCR

qRT-PCR was performed as described previously [26]. Bursa samples were frozen in liquid nitrogen and stored at −80 °C until RNA isolation. Tissue homogenization was done using a liquid nitrogen cooled steel mortar system and Trifast peqGOLD (Peqlab). Phase separation was achieved by addition of chloroform and the RNA was purified using the NucleoSpin RNA Kit (Macherey-Nagel). The quantity and purity of the received RNA was analyzed with the Nanodrop ND1000 system (Peqlab). A total of 100 ng RNA was transcribed into cDNA with the qScript cDNA Supermix (Quanta Biosciences). Expression levels of pro - and anti - inflammatory cytokines: IL1β, IL6, IL10, TNFα, TGFβ1 and MIF, markers for pain: COX2 and PENK, markers for innervation: GAP43 and PGP9.5, MMP1 and MMP9 and BMP2 were evaluated. qRT-PCR was performed with the SyBr Green Mastermix (Quanta Biosciences) according to the manufacturer’s manual and the LightCycler 480 System (Roche). All primer sequences were designed using Primer 3 software (http://frodo.wi.mit.edu/primer3) and the sequence of IL1β was taken from Brophy et al. [27]. Primer sequences are depicted in Table 3 and primers were produced by Tib MolBiol (Berlin, Germany). All primers were tested for amplification efficiency and an efficiency corrected equation was used to calculate the normalized expression to the reference gene 18S rRNA [28].

**Table 3:** qRT-PCR primer

Gene	Accession No.	Primer sequence
*18S rRNA*	NM_022551	Forward: 5′ CGGAAAATAGCCTTTGCCATC 3′ Reverse: 5′ AGTTCTCCCGCCCTCTTGGT 3′
*IL1b*	NM_000576	Forward: 5′ TCCAGGAGAATGACCTGAGC 3′ Reverse: 5′ GTGATCGTACAGGTGCATCG 3’
*TNFa*	NM_000594	Forward: 5’ AGCCCATGTTGTAGCAAACC 3′ Reverse: 5′ GAGGTACAGGCCCTCTGATG 3’
*IL6*	NM_000600	Forward: 5’ TGAGGAGACTTGCCTGGTGA 3′ Reverse: 5′ TTGGGTCAGGGGTGGTTATT 3’
*IL10*	NM_000572	Forward: 5’ TGAGAACAGCTGCACCCACT 3′ Reverse: 5′ GGCAACCCAGGTAACCCTTA 3’
*TGFb1*	NM_000660.4	Forward: 5’ AAGGACCTCGGCTGGAAGTG 3′ Reverse: 5′ AGGGCCAGGACCTTGCTGTA 3’
*MIF*	NM_002415	Forward: 5’ GGTTCCTCTCCGAGCTCACC 3′ Reverse: 5′ TAGACCCTGTCCGGGCTGAT 3’
*PGP9.5*	NM_004181	Forward: 5’ CCATACAGGCAGCCCATGAT 3′ Reverse: 5′ AGACCTTGGCAGCGTCCTTC 3’
*GAP43*	NM_002045	Forward: 5’ CCGGCAAAGCAGGAGAAACT 3′ Reverse: 5′ TGGAGGACGGCGAGTTATCA 3’
*COX2 (PTGS2)*	NM_000963	Forward: 5’ TAGAGCCCTTCCTCCTGTGC 3′ Reverse: 5′ TGGGGATCAGGGATGAACTT 3’
*PENK*	NM_001135690.3	Forward: 5’ TCCTGGCTTGCGTAATGGAA 3′ Reverse: 5′ TTTCTCTGAGGGTGCTGGTG 3’
*MMP1*	NM_002421.3	Forward: 5’ CACGCCAGATTTGCCAAGAG 3′ Reverse: 5′ GTCCCGATGATCTCCCCTGA 3’
*MMP9*	NM_004994.2	Forward: 5’ GGGACGCAGACATCGTCATC 3′ Reverse: 5′ GGGACCACAACTCGTCATCG 3’
*BMP2*	NM_001200.3	Forward: 5’ AGGAGGAGGCAAAGAAAAGG 3′ Reverse: 5′ GGAAGCAGCAACGCTAGA AG 3’

Abbreviations: *18S rRNA* ribosomal RNA, *IL1b* interleukin 1β, *TNFa* tumor necrosis factor α, *IL6* interleukin 6, *IL10* interleukin 10, *TGFb1* transforming growth factor β1, *MIF* macrophage migration inhibitory factor, *PGP9.5* protein gene product 9.5, *GAP43* growth associated protein 43, *COX2* cyclooxygenase 2, *PENK* proenkephalin, MMP1 Matrix Metalloproteinase 1, MMP9 Matrix Metalloproteinase 9, BMP2 Bone Morphogenetic Protein 2

### Statistics

Statistical analysis was performed using GraphPad Prism version 8.0.0. The Kruskal-Wallis Test followed by Dunn’s Multiple Comparison test was used to detect differences in the histological and gene expression data of the bursa tissue samples from the 4 shoulder pathology groups. A non-parametric Spearman’s Rho correlation (r_s_) analysis was performed to evaluate relationships between the SSP tear classification and the histological and gene expression data. The level of significance was set at *p* ≤ 0.05.

## Results

### Descriptive evaluation of bursa tissue

**Fig. 1:**
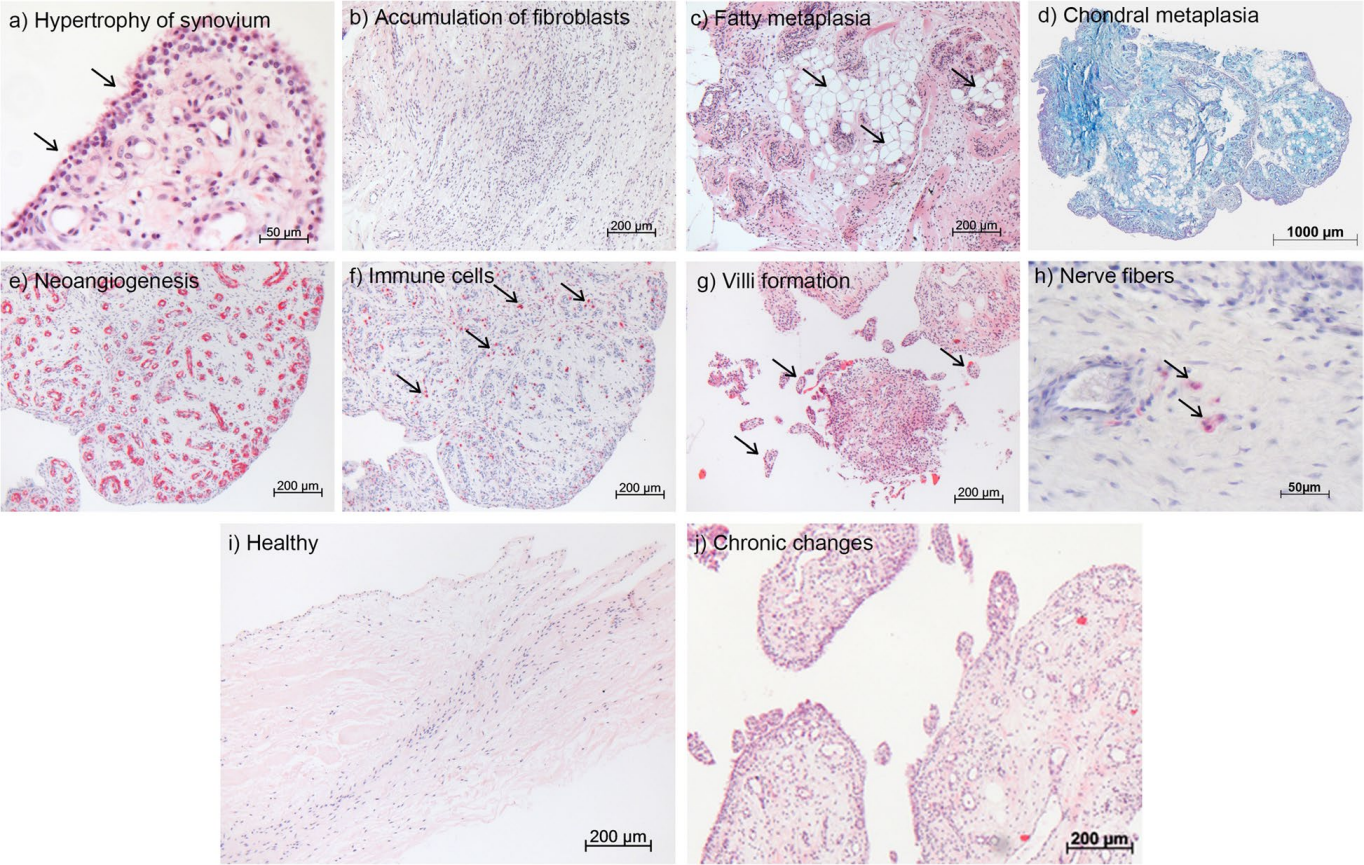
Representative images, taken from full-thickness tear group 4, of parameters evaluated via histological score (HS). **a** Hypertrophy of synovium (H&E staining). Up to 4 layers of synoviocytes are present, HS 3. **b** Number of fibroblasts with rounded nuclei (H&E staining) was greatly increased in the whole tissue, HS 3. **c** Fatty metaplasia (H&E staining), when locally detected, HS 2. **d** Chondral metaplasia (Alcian Blue staining) in entire tissue, HS 3. **e** Neoangiogenesis, as an indicator for new vessel formation (α-SMA staining, red signal), HS 3. **f** Number of immune cells (CD45 staining, red signal) inside the tissue and in high numbers, HS 3. **g** Occurrence of villi as a sign for chronic processes (H&E staining), HS 3. **h** Occurrence of few nerve fibers (S100 staining), HS 2. For comparison, a “healthy” bursa from group 1 is depicted in (**i**) and a bursa from full-thickness SSP tear group 4 undergoing chronic changes is depicted in (**j**) (H&E staining)

**Fig. 2:**
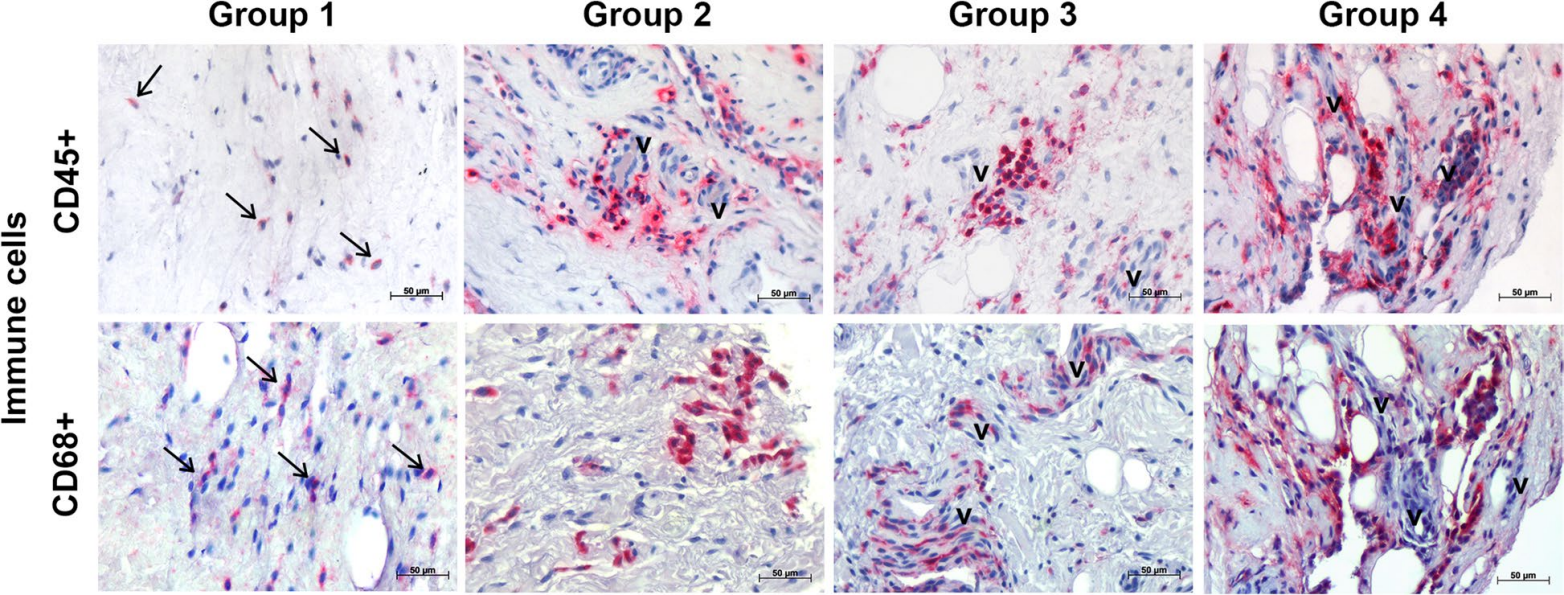
Representative immunohistological stainings of immune cells from the healthy control group 1, the osteochondral pathology group 2, the partial tear group 3 and the full-thickness tear group 4. The upper row shows CD45+ leukocytes, which are found mostly perivascular (v) in the 3 pathology groups and to a lesser extent in the healthy control group 1 (arrows). The lower row depicts CD68+ monocytes/macrophages, which are also more pronounced in pathological bursae (group 2–4) close to vessels (v) and sporadically detected in healthy bursae of group 1 (arrows). The scale bar represents 50 μm at 400x magnification

The histological scores described in Table 2 were used to characterize changes within the bursa tissue from the different shoulder pathology groups (Table 1). Examples of the investigated parameters are depicted in Fig. 1a-h. Healthy bursa tissue from group 1 showed an outer 1-cell layer of synoviocytes with an organized architecture of fibrous tissue underneath, consisting mainly of fibrocytes and a few vessels (Fig. 1i). Whereas bursae from pathology groups 2, 3, and 4 displayed a multilayered synovial membrane, high numbers of fibroblasts, vessels, villi and nerve fibers, as well as chondral metaplasia (Fig. 1a-h). CD45+ leukocytes were present mostly in perivascular spaces in all 3 pathology groups. To a lesser extent, leukocytes were also found in the healthy control group. The CD45+ leukocytes included high proportions of CD68+ monocytes/macrophages. Similarly, in the pathology groups more monocytes/macrophages are present, which accumulate particularly close to blood vessels. In healthy controls CD68+ cells can be found more sporadically within the tissue (Fig. 2). Altogether, bursae from these three pathology groups were considered to have undergone morphological changes towards a degenerative phenotype as depicted in Fig. 1j.

### Quantitative histological evaluation of bursa tissue

**Fig. 3:**
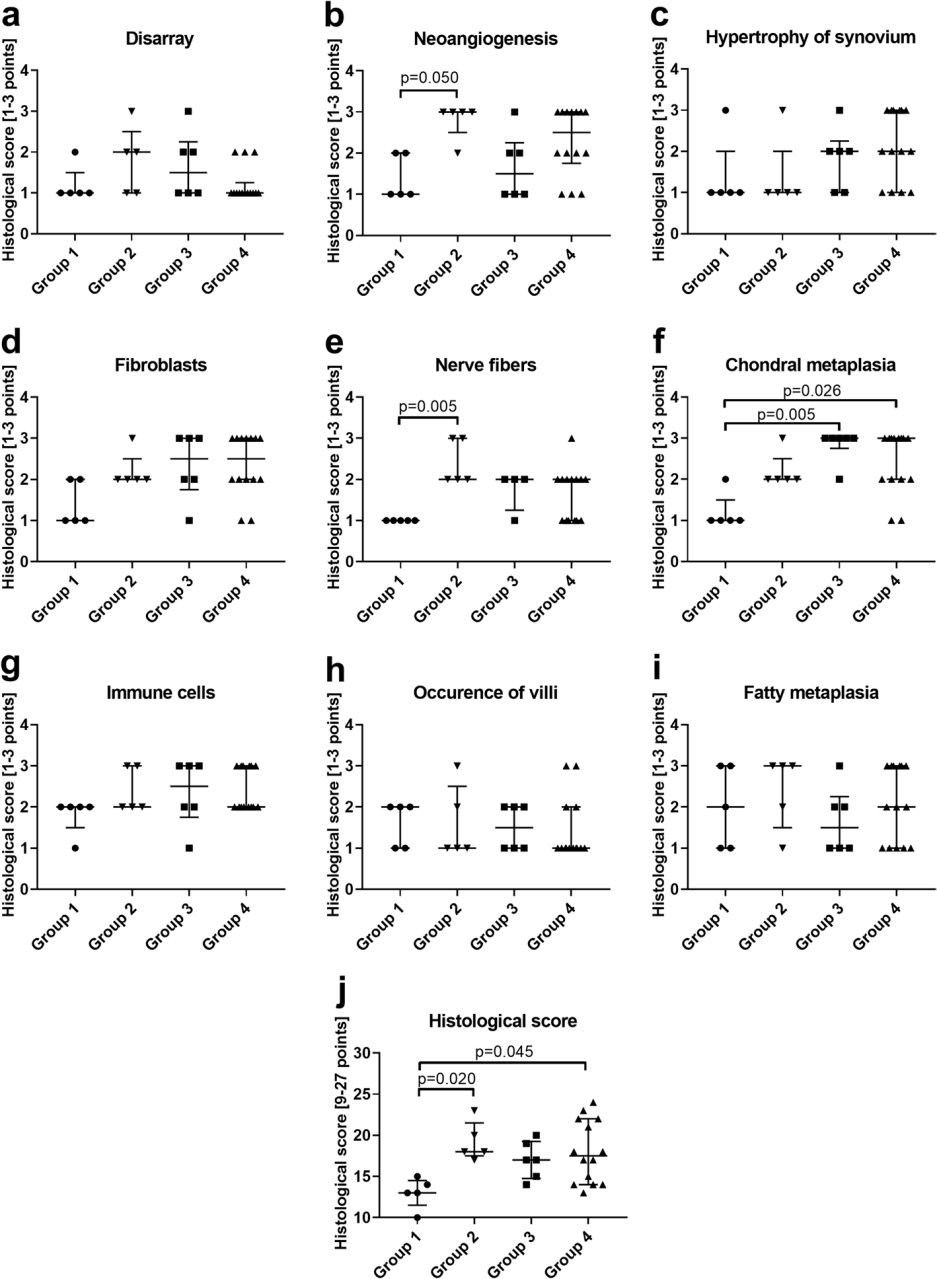
Evaluation of histological score (1: normal/absent, 2: Focal increase, 3: highly altered/extended) in bursae of healthy group 1 (*n* = 5), group 2 with osteochondral pathology without RC tear (*n* = 5), group 3 with partial SSP tear (*n* = 6) and group 4 with full-thickness SSP tear (*n* = 14). A minimum of 9 to a maximum of 27 points could be reached. **a** Disarray of tissue. **b** Neoangiogenesis. **c** Hypertrophy of synovium. **d** Number of fibroblasts. **e** Nerve fibers. **f** Chondral metaplasia. **g** Number of immune cells. **h** Occurrence of villi. **i** Fatty metaplasia. **j** Total histological score was analyzed with a 3-point system of 9 parameters reaching a maximum of 27 points, which would indicate a highly altered bursa tissue. Data are presented as individual dot plots with median and interquartile range. Statistics: Dunn’s Multiple Comparison Test; *p* ≤ 0.05

Bursa samples from group 1 (healthy controls) showed the lowest overall histological score with a median of 13 points (range 10–15) with the lowest scores for disarray, vessels, hypertrophy of synoviocytes, fibroblasts, nerve fibers, and chondral metaplasia (Fig. 3a-f, j). Immune cells were present at low numbers and located mostly in perivascular spaces (Fig. 3g). Fatty metaplasia and villi formation was scored similar compared to the three pathology groups (Fig. 3h, i). Bursae from group 2 with degenerative osteochondral pathology showed a significantly enhanced total histological score of 18 (range 17-23) points compared to healthy group 1 (*p* = 0.02; Fig. 3j). The characteristics of these bursae manifested in significantly higher numbers of vessels and nerve fibers compared to group 1 (*p* = 0.05 and *p* = 0.005 respectively, Fig. 3b, e). Bursae from group 3 with partial SSP tear showed an increased histological score with a median of 17 (range 14-20) points with significantly increased chondral metaplasia compared to group 1 (*p* = 0.005; Fig. 3f, j). Bursae from group 4 with full-thickness SSP tear displayed a significantly increased total histological score of 17.5 (range 13-24) points compared to group 1 (*p* = 0.045). This was caused by significantly increased chondral metaplasia (*p* = 0.026) extended angiogenesis, a frequently thicker synovial layer, and higher numbers of immune cells and fibroblasts (Fig. 3b, c, d, f, g, j). Altogether, significant differences in the quantitative histological evaluation occurred only between the healthy group 1 and the 3 pathology groups, but not between the pathology groups. The individual parameters only partially differed between the groups regarding angiogenesis, presence of nerve fibers, and chondral metaplasia. However, the combination of parameters in the total histological score underlines bursal tissue alterations towards a degenerative phenotype in patients with SSP tear and osteochondral defects.

### Gene expression analysis

**Fig. 4:**
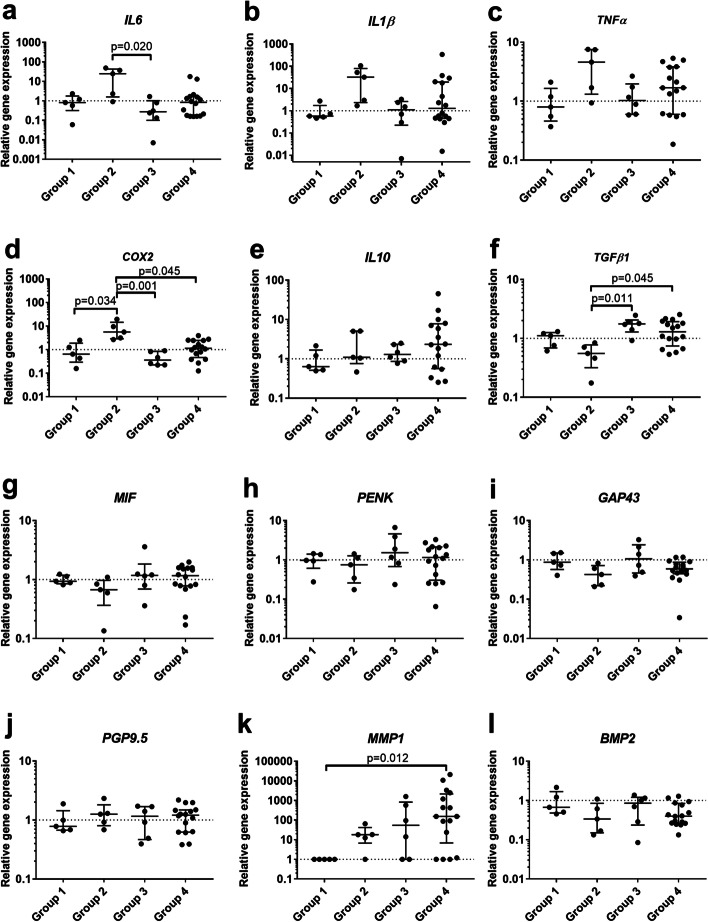
Relative gene expression of pro- and anti-inflammatory factors and nerve markers in bursae of healthy group 1 (*n* = 5), group 2 with osteochondral pathology without RC tear (*n* = 5), group 3 with partial SSP tear (*n* = 6) and group 4 with full-thickness SSP tear (*n* = 16). qRT-PCR data were normalized to the expression of the house keeping gene 18S rRNA. The data are shown as fold values to the healthy group (dashed line) and given as individual dot plots with median and interquartile range. For MMP1 no expression was detected in the healthy group 1 and values were set to a fold of 1. Statistics: Dunn’s Multiple Comparison Test

Pro-inflammatory cytokines such as IL6, IL1β, TNFα and COX2 were enhanced in group 2 with degenerative osteochondral pathology, with significant differences in IL6 expression compared to group 3 (*p* = 0.020) and for COX2 expression compared to groups 1, 3 and 4 (*p* = 0.034, *p* = 0.001, *p* = 0.045 respectively) (Fig. 4a-d). IL10 expression highly varied in group 4 with full-thickness SSP tear and showed no significant differences between the groups (Fig. 4e). Expression of TGFβ1 was significantly elevated in the SSP tear groups 3 and 4 compared to group 2 (*p* = 0.011, *p* = 0.045, respectively; Fig. 4f). The nerve and pain associated markers MIF, PENK, GAP43, and PGP9.5 were not differentially regulated in any of the pathology groups analyzed (Fig. 4g-j). MMP1 expression was not detectable in the healthy group 1, but was markedly upregulated in the pathology groups with a significant increase in the bursae of the full-thickness SSP tear group 4 (*p* = 0.012). Standard deviation prevented statistical significance in group 2 and 3, as also 1 donor in group 2, 2 donors in group 3 and 3 donors in group 4 showed no MMP1 expression (Fig. 4k). MMP9 was not expressed in any of the investigated groups and BMP2 expression was unchanged between the groups (Fig. 4l). Altogether, the gene expression analysis enabled the detection of group differences in gene expression between the different pathology groups.

### Correlation analysis

For the groups with partial and full-thickness SSP tears (group 3 and 4) a correlation analysis between the extent of the tear according to Patte and Bateman classification and the histological and molecular biological data was performed. The Patte classification for tendon retraction positively correlated with the IL1β and COX2 expression (r_s_ = 0.422, *p* = 0.050; r_s_ = 0.542, *p* = 0.009, respectively) and the Bateman classification for tear size positively correlated with the expression of IL6 and COX2 (r_s_ = 0.427, *p* = 0.047; r_s_ = 0.541, *p* = 0.015, respectively). No correlations occurred between the extent of the tear and the histological characteristics. The positive correlation of the clinical scores with the expression of the inflammatory markers show an increase of pro-inflammatory mediators with the severity of SSP tear.

## Discussion

This pilot study provides structural and molecular characteristics of bursa tissue from varying shoulder pathologies. We aimed to determine specific tissue alteration of the subacromial bursa according to RC pathologies compared to non-tendon defects of the shoulder. Therefore, biopsies taken from the subacromial bursa from healthy patients, patients with osteochondral pathologies without RC tear or with degenerative tears of the RC were investigated. In summary, the bursae of the osteochondral and full-thickness SSP tear groups histologically showed the strongest tissue alteration compared to the healthy control group. Increased MMP1 expression seems to be an important marker for a bursal reaction in pathological shoulders and particularly full-thickness SSP tears. Additionally, expression of pro-inflammatory cytokines correlated with the severity of the SSP tear. Beside these distinct differences, alterations on the molecular level between the pathology groups were small.

Bursae from patients with shoulder or AC joint instability (group 1) had a low overall histological score and resembled healthy tissue. When compared to healthy bursae, tissue from the pathology groups 2–4 scored higher in the histological score classification system with respect to the number of fibroblasts, blood vessels and nerve fibers present. These results are in accordance with previous findings, showing that altered bursa tissue is well vascularized [12] and a rich source of cells such as fibroblasts, synoviocytes, endothelial cells and immune cells [13, 29]. The present study supports the presence of leukocytes and monocytes/macrophages in bursae of pathological shoulders. However, also in bursae of healthy shoulders immune cells were detected in lower amounts. The histological results indicate that the bursa structure changes with degenerative shoulder pathologies independent of the occurrence of an SSP tear. Chondral metaplasia was high in all three pathology groups but significantly increased in the SSP tear groups 3 and 4 compared to the healthy control group. Chondral metaplasia appears to be a marker for chronic degeneration, as it is also found in tendinopathic Achilles tendons, but less so in acute ruptures [30]. Whether the chondral metaplasia in the bursa tissue is the cause or consequence of the SSP tear is still open to debate. One could speculate that increased chondral metaplasia may cause changes in bursa stiffness possibly resulting in a limited friction-reducing capacity of the bursa. This might lead to degenerative changes at the tendon interface, which could subsequently promote tendon tearing. For SSP tendon but not bursa tissue a negative correlation between chondral metaplasia and tendon healing has been described [24]. Previously, the bursa was claimed as possible reason for tendon tearing justified by the presence of active BMPs in the bursa, which might induce ectopic bone/cartilage formation [31]. Presently the investigation of BMP2 expression confirmed the presence of BMPs in the bursa, but did not provide any evidence of its negative contribution in tendon tearing, due to unchanged expression levels between the groups. The occurrence of nerve fibers was significantly elevated in bursae from group 2 with osteochondral pathology and also partially increased in the SSP tear groups 3 and 4 compared to healthy group 1 in which no nerve fibers were detectable. In contrast to the histological findings, the gene expression analysis of nerve and pain associated markers revealed no significant differences between the groups, which might be explained by high patient specific variations. However, the histological results may support findings from other studies in which the bursa is suggested to be the source of shoulder pain [7, 32, 33]. Pain scores were not routinely evaluated in this study and therefore we can only speculate on the correlation between pain and the occurrence of nerve fibers.

Both SSP tears and osteochondral pathology are longstanding chronic pathologies, however the present RNA analysis revealed differences in the inflammatory status of bursa tissue from these patients, which might be due to interaction between the bursa and the synovial fluid in case of RC tear. This interaction is only possible through the open compartment between the subacromial space and the glenohumeral joint in case of an RC tear. A relationship between the extent of RC tears or pain with the expression of markers in the synovial fluid has been shown previously and next to IL1β, MMPs seem to play an important role as possible biomarkers for RC tears [34, 35]. The strongly increased MMP1 levels in the present full-thickness SSP tear group underlines this possible interaction between the subacromial bursa and synovial fluid. As expression levels of inflammatory markers for partial and full-thickness SSP tears were mostly similar, the bursa-synovia interaction might not fully explain the changes. The present result of highly increased MMP1 expression in bursae of full-thickness SSP tears strongly supports other studies showing comparable results [7, 36]. This increase in MMP1 expression hints for a strong bursal reaction with a highly upregulated remodeling process in bursae from pathological shoulders and particularly SSP tears. The expression of the pro-inflammatory cytokines IL6, TNFα and COX2 were significantly increased in bursae from group 2 with osteochondral pathology, whereas TGFβ1, an immune modulator with inflammation resolving potential [37], was significantly decreased. This might indicate sustained inflammatory reactions in bursae of patients with osteochondral defects. Additionally, others have previously shown that pro-inflammatory factors and cytokines are increased in patients with RC tear versus patients with shoulder instability [7, 13, 32, 38]. In contrast, we found no significant elevation in expression of pro-inflammatory markers in bursae from patients with SSP tears when compared with the control group. Together with the presence of immune cells in bursae of the healthy group 1, this might indicate that even in healthy patients the subacromial bursa serves as an inflammatory reservoir. However, our correlation analysis showed that expression of inflammatory mediators such as IL6, IL1β, and COX2 increased with the severity of the RC tear, thus indicating that bursa tissue from patients with higher severity of SSP tears is characterized by an increased pro-inflammatory status. The variability of tear severity within the SSP tear groups may have masked potential differences when compared to the control group, hence no significant group differences in inflammatory gene expression were observed. Furthermore, Chillemi et al. reported that bursal tissue inflammation decreases over time, meaning that the longer the time period after the onset of symptoms of an RC lesion, the lower the acute inflammatory reactions in the tissue might be [24]. Therefore, the differences in time between the onset of symptoms and patient surgery might also have affected the results in terms of inflammatory gene expression. A correlation analysis of the investigated parameters with the duration of tear pathology could have clarified this. However, the patient data for such analysis was not available. In addition to the anti-inflammatory potential of TGFβ, the growth factor has been shown to have contradictory functions in tendon healing. On the one hand, TGFβ is required in the normal tendon healing process [39] and its over-expression in bone marrow stem cells (BMSC) enhanced tendon to bone healing in a rabbit anterior cruciate ligament model [40]. On the other hand, TGFβ is associated with scarring and amongst other growth factors it is a key mediator in the pathogenesis of fibrosis [41]. Furthermore, increased TGFβ1 expression has been linked to increased numbers of chondrocytes in the ACL [40], which could explain the stronger chondral metaplasia observed in the bursa samples with increased TGFβ expression in the present study. Without further investigation, the contradictory functions of TGFβ make it difficult to clearly state the effects of its expression in bursa tissue on tendon healing processes.

We have to acknowledge the limitations of this pilot study. Demographic data such as the age or sex of the patients were not considered in the analysis as an independent confounder, which is justified by the automatically underlying age difference of the various shoulder pathologies. Therefore, differences between the healthy group 1 and the degenerative groups 2–4 could partially result from age or sex related changes. We speculate that differences between the full-thickness SSP tear group and the other groups might result from interaction with synovial fluid. An analysis of the synovial fluid of the patients would help to support this hypothesis but was unfortunately not available. As a further limitation, surgical techniques differed between the groups, as patients from group 2 were treated with an open surgical technique, whereas all other patients were treated arthroscopically. The more invasive removal of biopsies could have an effect on the results obtained. Furthermore, the group sizes in this pilot study differed due to the availability of the human bursa material, which additionally may have affected observed statistical differences.

## Conclusion

In contrast to tendons, bursa tissue is well vascularized and a source of potentially beneficial cell populations for tissue healing processes, including stem cells and immune cells. We have demonstrated that characteristics of bursa tissues differ depending on the shoulder pathology. Especially, a strongly increased MMP1 expression seems to be a marker for full-thickness SSP tears, whereas the expression of the pro-inflammatory mediators IL1β, IL6, and COX2 increased with the severity of the SSP tear. Additionally, both SSP tear groups exhibited increased chondral metaplasia and TGFβ1 expression. Bursae from patients with osteochondral pathology were characterized by increased angiogenesis and nerve fibers as well as increased IL6 and COX2 expression. Despite the clear differences in the underlying pathology of the patient groups, the differences were not as pronounced as expected, which indicates that the bursa structure alters with degenerative shoulder pathologies independent of the occurrence of an SSP tear. Additionally, even bursae of heathy donors seem to be a reservoir for inflammatory cells and mediators. We believe the histological and molecular characterization detailed in this pilot study contributes to the understanding of structural and molecular tissue alterations related to RC tears or non-tendon shoulder pathologies, paving the way for future studies further elucidating the role of the subacromial bursa in the development or healing of shoulder pathologies.

## Data Availability

The datasets analyzed during the current study are available from the corresponding author on reasonable request.
